# Ultrasound-detected subclinical inflammation was better reflected by the disease activity score (DAS-28) in patients with suspicion of inflammatory arthritis compared to established rheumatoid arthritis

**DOI:** 10.1007/s10067-016-3326-6

**Published:** 2016-06-21

**Authors:** Coziana Ciurtin, Karol Wyszynski, Robert Clarke, Maria Mouyis, Jessica Manson, Giampiero Marra

**Affiliations:** 1 Department of Rheumatology, University College London Hospitals NHS Trust, 3rd Floor Central, 250 Euston Road, London, NW1 2PG UK; 2 Department of Statistics, University College London, London, UK; 3 Medical School, University College London, London, UK; 4 Department of Rheumatology, Northwick Park Hospital, Harrow, UK

**Keywords:** Inflammatory joint pains, Joint ultrasound, Prediction of power Doppler, Rheumatoid arthritis, Subclinical inflammation

## Abstract

Limited data are available about the ultrasound (US)-detected inflammatory features in patients with suspicion of inflammatory arthritis (S-IA) vs. established rheumatoid arthritis (RA). Our study aimed to assess if the presence of power Doppler (PD) can be predicted by a combination of clinical, laboratory and US parameters. We conducted a real-life, retrospective cohort study comparing clinical, laboratory and US parameters of 108 patients with established RA and 93 patients with S-IA. We propose a PD signal prediction model based on a beta-binomial distribution for PD variable using a mix of outcome measures. Patients with RA in clinical remission had significantly more active inflammation and erosions on US when compared with patients with S-IA with similar disease scores (*p* = 0.03 and *p* = 0.01, respectively); however, RA patients with different disease activity score (DAS-28) scores had similar PD scores (*p* = 0.058). The PD scores did not correlate with erosions (*p* = 0.38) or DAS-28 scores (*p* = 0.28) in RA patients, but they correlated with high disease activity in S-IA patients (*p* = 0.048). Subclinical inflammation is more common in patients with RA in clinical remission or with low disease activity than in patients with S-IA; therefore, US was more useful in assessing for true remission in RA rather than diagnosing IA in patients with low disease activity scores. This is the first study to propose a PD prediction model integrating several outcome measures in the two different groups of patients. Further research into validating this model can minimise the risk of underdiagnosing subclinical inflammation.

## Introduction

Ultrasound (US) assessment of small joints is routinely used for the diagnosis of peripheral inflammatory arthritis (IA) and helps guiding therapeutic decisions in patients with established rheumatoid arthritis (RA) [1]. The access to musculoskeletal US services varies among hospitals and rheumatology services. The usefulness of the US examination for the diagnosis and management of RA depends on the level of expertise of examiners and quality of the US machines [2]. Even if a considerable proportion of patients with established RA continue to have subclinical inflammation, despite evidence of clinical remission, it is not cost-effective to screen them all. Extensive US examination of peripheral joints in RA had a good predictive value for disease outcome, as established by an 18-month longitudinal study [3]. As the US examination of multiple joints can be time consuming, several US scoring systems have been developed, aiming to assess a smaller number of joints without compromising on the quality of data collected [4].

There are no guidelines to help us decide which RA patients should have an US scan of their joints or how often, despite the constant effort to generate recommendations regarding the use of imaging techniques in the management of patients with RA [5]. There is no straightforward indicator of the risk of continuing with active joint inflammation, despite the use of several US prediction factors for disease progression and damage [6–8].

The OMERACT (Outcome Measures in RA Clinical Trials) initiative defined the US abnormalities characteristic for RA as synovial hypertrophy (SH), with or without power Doppler (PD) signal, tenosynovitis and erosions [4, 9].

The available US scoring systems use different quantitative or semiquantitative measures (such as grades of SH or PD) or a binary scoring system (such as presence/absence of erosions) to express US findings [4]. The principal aim of the international and European US expert groups is to develop a standardised US scoring system which will capture the patient’s global disease activity and which can be employed to guide therapeutic decisions [10].

As several outcome measures are required to establish if patients with RA have active disease or not, we identified the need to integrate clinical and laboratory parameters in a prediction model that could improve the quality of care we provide to our patients, by enabling the identification of those at risk of having positive PD signal in their joints. In an ideal situation, patients with a previous diagnosis of RA or with the suspicion of having developed IA are offered an US examination of their joints to increase the chance of correct diagnosis and optimise disease control. In reality, because of limited resources, patients are referred to US clinics in a selective manner, based on their clinician expertise and need, and availability of US resources. However, it is widely recognised that subtle joint inflammation is often missed by the clinical examination [11]. It was proposed that RA treatment should target the control of sub-clinical inflammation (as assessed by US or MRI), instead of being exclusively guided by clinical examination and laboratory measures [12], and that remission criteria for patients with RA should also include joint US examination [13, 14].

The rationale of our research project was to generate useful information for clinicians that can be easily applied in real life and can help identify patients at risk of having active inflammation in their joints. Our statistical modelling framework is based on data routinely collected from a heterogeneous RA population.

The aim of our study was to build a statistical model to assess the influence of several outcome measures on the presence of PD signal. The selected outcomes were as follows: the number of tender joints (TJC) and swollen joints (SJC) out of 28, global health state as assessed using a visual analogue scale (GVAS), high-sensitivity C-reactive protein (hsCRP), erythrocyte sedimentation rate (ESR), presence of rheumatoid factor (RF) and anti-citrullinated cyclic peptides antibodies (ACPA) and anti-nuclear antibodies (ANA), disease duration and medication. In order to generate this prediction model, we collected similar data from the two groups of patients (with suspicion of inflammatory arthritis (S-IA) and RA) and assessed the influence of every parameter on the patients’ risk of having active PD in their joints.

## Methods

### Subjects

We conducted a real-life, retrospective cohort study of patients seen to our US outpatient clinics in the order of their referral, between May 2013 and September 2013. We used an established protocol of US examination of hands comprising flexor tendons and 22 joint assessments (dorsal longitudinal and transverse views), which is the standard of practice for our US clinics. All the patients were referred to this clinic for inflammatory symptoms in their hands, and their clinical examination was equivocal. No inclusion or exclusion criteria were applied for the selection of patients, to ensure the general applicability of the prediction model we propose. Patients on oral steroids or NSAIDs, or who had steroid injections less than 4 weeks before the US scan, were excluded from the final analysis. We report data on the two largest groups, the patients with established RA and patients with S-IA, using descriptive statistics (SPSS, version 22). We also stratified patients based on their disease activity assessed using the disease activity score assessing 28 joints (DAS-28). Patient groups with different DAS scores were compared using ANOVA/chi-squared test depending on the variables analysed (*p* < 0.05 was considered significant). We also performed a cluster analysis of patients with similar DAS-28 scores using the aforementioned outcome measures.

### Ultrasound examination

US examination was performed using a Logiq S8 US machine (GE Medical Systems Ultrasound and Primary Care Diagnostics, Wauwatosa, WI, USA), equipped with a multi-frequency linear matrix array transducer (6–15 MHz). B-mode and PD machine setting are optimised and standardised for all our patients’ US examinations. The settings used were as follows: B-mode frequency 11–15 MHz and Doppler frequency 7.5–15 depending on the depth of the anatomical area, Doppler gain around 18 dB, low wall filters and pulse repetition frequency 800 Hz. Because of the small number of joints with PD signal (only patients with equivocal clinical examination were referred for an US examination of their hands), we did not report separately the grades of PD signal.

### Statistical analysis for the prediction model

We proposed a regression model to assess the contribution of every outcome measure to the risk of having active joint inflammation as well as predict PD signal. We excluded patients with PD signal present in more than 10/22 joints to ensure homogeneity in the data. We conducted a real-life study including 276 patients referred for the suspicion of active joint inflammation (new referrals for S-IA, RA patients and patients with other inflammatory rheumatic conditions). We assessed 22 hand joints in every patient, irrespective of their hand symptoms, using the OMERACT scoring system for US examination. The proposed regression model was based on a beta-binomial distribution (with the response ranging from 0 to 10) for the PD score variable and a mix of main interaction effects for the outcome measures stated earlier. Negative interaction effects show that the respective outcome is associated with a lower number of joints with PD signal.

## Results

We collected data from 276 consecutive patients referred to our rheumatologist-led US clinic to have a scan of their hand joints aiming to answer the clinician question about the presence of active inflammation in their joints. There were 108 patients with established RA, 93 were referred for the clinical suspicion of IA, 29 were previously diagnosed with psoriatic arthritis (PsA) and 46 patients had other diagnoses, including crystal arthropathies, sarcoidosis, osteoarthritis and chronic pain. We analysed in parallel the characteristics of the two main groups of patients: the group of established RA (*n* = 108) and the group of patients referred with S-IA (*n* = 93) (Table 1). The assessment of the follow-up clinical letters of S-IA patients revealed that 50.5 % (*n* = 47) were ultimately diagnosed with a form of IA, out of which 76.5 % (*n* = 36) were diagnosed with RA, 12.7 % (*n* = 6) with undifferentiated IA and 10.6 % (*n* = 5) with psoriatic arthritis. The rest of the patients initially referred with the suspicion of IA (49.4 %) were subsequently diagnosed with non-specific arthralgia, chronic pain or hand osteoarthritis.

**Table 1:** Comparison between patients with S-IA and established RA

Disease	S-IA (*n* = 93)	RA (*n* = 108)	*p* value
Mean age ± SD	50.69 ± 15.7	55.9 ± 15.2	0.04
Sex (%)	87.1 % F (*n* = 81)	79.6 % F (*n* = 86)	0.73
Duration of the symptoms	6.8 months ± 1.9 Median = 7 months IQR = 13 months	Median = 60 months IQR = 108	0.001
% (*n*) of patients on NSAIDs and painkillers at the time of the scan	0.096 % (9/93)	0.194 % (21/108)	0.023
% (*n*) of patients on steroids at the time of US scan	0.01 (1/93)	0.05 (6/108)	0.055
Mean dose	10 mg oral prednisolone	120 mg DepoMedrone i.m. (given 5 ± 1 week ago).	
Number of days since given steroids to the time of US scan	One patient on oral prednisolone at the time of the scan	Mean = 19.3 ± 15.73 days Median = 19 IQR = 21.25	N/A
hsCRP (mean ± SE)	6.92 ± 1.4	5.57 ± 0.76	0.38
Median and IQR	Median = 2.7 IQR = 5.65	Median = 2.9 IQR = 5.95	0.58
ESR (mean ± SE)	17.2 ± 16.3	20.6 ± 18.8	0.62
Median and IQR	Median = 12 IQR = 18	Median = 14 IQR = 22.75	0.76
% patients who had the blood tests taken within 2 weeks from the time of the US scan	98 % (92/93)	84.4 % (92/109)	0.009
% RF positive	25.8 % (24/93)	67.6 % (73/108)	0.007
% ACPA positive	24.7 % (22/89)	83 % (90/108)	0.001
Number of joints with SH grade 1 (median and IQR)	Median = 1.5 IQR = 2.25	Median = 2 IQR = 4.5	0.54
Number of joints with SH grade 2 (median and IQR)	Median = 0 IQR = 3	Median = 1 IQR = 3	0.025
Number of joints with SH grade 3 (median and IQR)	Median = 0 IQR = 0	Median = 2 IQR = 4.25	0.013
Number of joints with PD (median and IQR)	Median = 0 IQR = 1	Median = 0 IQR = 2.25	0.03
Number of joints with osteophytes (median and IQR)	Median = 1 IQR = 5.25	Median = 1 IQR = 4	0.23
Number of joints with erosions (median and IQR)	Median = 0 IQR = 4.5	Median = 2 IQR = 8.75	0.004
% (*n*) patients with PD	55.9 % (52/93)	41.6 % (45/108)	0.56
TJC (mean ± SD)	8.81 ± 9.07 Median = 5.5	9.54 ± 8.9 Median = 6	0.57
SJC (mean ± SD)	2.66 ± 5.18 Median = 1	3.73 ± 4.74 Median = 2	0.12
GVAS (mean ± SD)	61.7 ± 25.7 (*n* = 75)	50.7 ± 28.9 (*n* = 105)	0.009
Pain VAS (mean ± SD)	59.9 ± 26.6 (*n* = 48)	53.3 ± 31.02 (*n* = 60)	0.24

The patients in the RA group were treated with methotrexate (MTX) alone or in combination with other synthetic disease-modifying anti-rheumatic drugs (DMARDs) in 79.6 % (86/108), SSZ alone or in combination in 48.1 % (52/108) and HCQ alone or in combination in 58.3 % (58/108). In terms of biologic treatments, 24.07 % (26/108) were on etanercept, 12 % (13/108) on adalimumab, 0.018 % on certolizumab (2/108), 0.09 % on rituximab (10/108) and 0.055 % on tocilizumab (6/108). Out of 108 patients with established RA, 89 patients (82.4 %) were on combination therapy. The RA patients were on stable conventional and biologic DMARD medication for at least 1 month prior to the scan. A minority of patients (21/108 in the RA group and 9/93 in the S-IA group) were treated with additional NSAIDs, and one S-IA had oral steroids (Table 1). These patients were excluded from the further analysis that stratified them based on their disease activity.

The comparison between the RA and S-IA groups with regard of the demographic, clinical, laboratory and US features is summarised in Table 1. The median number of joints with PD activity was significantly higher in RA patients (0, interquartile range (IQR) = 2.25 vs. 0, IQR = 1, *p* = 0.03), as was the number of joints with erosions (*p* = 0.004) and the number of joints with joint effusions (1.5 vs. 0, *p* = 0.013). However, the percentage of patients having at least one joint with active inflammation, as assessed by the presence of PD signal, was not significantly different between the two groups (52/93, 55.9 % vs. 45/108, 41.6 %, *p* = 0.56). Tendon abnormalities (such as effusion and tendon sheet thickening) were more frequently observed in patients with RA vs. S-IA (14/108 vs. 3/93, *p* = 0.042). The number of clinically assessed swollen joints correlated poorly with the US-detected inflammation (as assessed by PD signal) in both groups of RA and S-IA patients (*r* = 0.27, *p* = 0.54 and *r* = 0.39, *p* = 0.72, respectively).

The majority of patients with S-IA and positive PD signal in at least one joint (*n* = 52) were subsequently diagnosed with a type of inflammatory arthritis (47/52, 90.3 %), out of which, 36 patients were diagnosed with RA (69.2 %). In five cases, the presence of PD was associated with osteophytes and they have been diagnosed with inflammatory OA. Out of 25.8 % of patients with S-IA who had positive RF at the time of the US scan (*n* = 24/93), 23 patients were subsequently diagnosed with RA (23/36, 63.3 %); similarly, a proportion of ACPA-positive S-IA patients were ulterior diagnosed with RA (18/36, 50 %).

Further analysis compared RA patients stratified based on the DAS-28 activity scores (Table 2), and similarly, Table 3 comprises data on the S-IA group (Table 3). As mentioned earlier, the patients on oral steroids and NSAIDs were excluded from this analysis, which reported data on 87 RA and 82 S-IA patients. As expected, the RA patients stratified based on their DAS-28 scores had statistically different ESR, TJC, SJC, GVAS and pain scores; however, they had similar US parameters assessing for active and chronic inflammatory changes (Table 2). In contrast, the analysis of patients with S-IA revealed that DAS-28 score stratification identified patients with different PD and SH grade 3 scores, CRP and ESR levels, as well as patients with different TJC, SJC, GVAS and pain scores (Table 3). In addition, the S-IA groups with low, moderate and active diseases had a larger proportion of patients with true active inflammation (based on PD signal) than the group classified as being in clinical remission (*p* = 0.01) (Table 3). The comparison between the patient groups with a similar disease activity revealed that patients with RA and moderate and high disease activities (DAS-28 = 3.2–5.1 and >5.1, respectively) were significantly different from S-IA patients with similar DAS-28 scores, as far as the disease duration, ACPA positivity and number of joints with erosions were concerned (Table 4). RA patients in remission (DAS-28 < 2.6) had significantly higher TJC, number of joints with active inflammation and erosions, and higher number of patients with subclinical inflammation than the S-IA patients classified as being in clinical remission (Table 4). We also assessed for correlations between PD signal and erosions in RA patients in all the disease activity groups, and there were no positive correlations (*p* = 0.06, *p* = 0.26, *p* = 0.49, *p* = 0.12 for the different DAS-28 score groups, and *p* = 0.38 for the whole RA group, respectively). Out of all clinical outcomes, only SJC correlated with the PD score and only in patients with RA (*p* = 0.03). The presence of erosions also correlated with the patient’s age and disease duration (*p* = 0.04 and *p* = 0.03, respectively).

**Table 2:** Comparison between RA patients with different levels of disease activity

Disease activity	RA (*n* = 11) DAS-28 <2.6	RA (*n* = 9) DAS-28 = 2.6–3.2	RA (*n* = 39) DAS-28 = 3.2–5.1	RA (*n* = 28) DAS-28 >5.1	ANOVA/chi-squared test
Mean age ± SD	47.08 ± 19.64	46.33 ± 12.59	58.17 ± 16.07	57.36 ± 15.69	0.051
Sex (% female)	0.67	0.56	0.65	0.70	0.885
Duration of the symptoms	7.92	6.71	11.44	8.31	0.300
hsCRP (mean ± SE)	1.56 ± 0.58	3.63 ± 2.13	5.49 ± 1.04	7.63 ± 1.73	0.127
Median and IQR	0.8 (0, 2.08)	0.9 (0, 3.2)	3.2 (1.1, 7.92)	4.4 (1.8, 11.1)	
ESR (mean ± SE)	7.5 ± 1.38	9.78 ± 2.52	19.44 ± 2.60	30.33 ± 3.46	0.000
Median and IQR	5 (5, 9.25)	7 (5, 13)	13.5 (7, 25.25)	32 (12, 37)	
% RF positive	0.5	0.67	0.58	0.55	0.881
% ACPA positive	0.5	0.56	0.42	0.55	0.690
Number of joints with SH grade 1 (median and IQR)	0 (0, 0.5)	0 (0, 0.5)	0 (0, 8.5)	0 (0, 2)	0.365
Number of joints with SH grade 2 (median and IQR)	0 (0, 0)	0 (0, 0)	0 (0, 0)	0 (0, 0)	0.836
Number of joints with SH grade 3 (median and IQR)	1 (0, 3.25)	0 (0, 3)	2 (0, 4.25)	3 (0, 5)	0.639
Number of joints with PD (median and IQR)	0.5 (0, 1)	0 (0, 1)	1 (0, 3)	1 (0, 4)	0.058
Number of joints with osteophytes (median and IQR)	0 (0, 5.75)	6 (0, 7)	3.5 (0, 8)	4 (0, 7)	0.993
Number of joints with erosions (median and IQR)	3.5 (1, 6.25)	4 (2, 4)	7 (2, 10)	5 (1, 11)	0.341
% patients with PD	0.5	0.33	0.63	0.67	0.257
TJC (mean ± SD)	1 ± 1.21	2.56 ± 1.81	6.51 ± 4.98	19.48 ± 7.78	0.000
SJC (mean ± SD)	1.75 ± 5.74	1.22 ± 1.92	4.98 ± 3.35	5.94 ± 5.09	0.002
GVAS (mean ± SD)	18.42 ± 24.74	34.44 ± 18.73	44.81 ± 25.44	74.55 ± 17.74	0.000
Pain VAS (mean ± SD)	11.42 ± 13.45	42 ± 26.12	49 ± 26.57	80 ± 19.10	0.000

**Table 3:** Comparison between S-IA patients with different levels of disease activity

Disease activity	S-IA (*n* = 16) DAS-28 <2.6	S-IA (*n* = 5) DAS-28 = 2.6–3.2	S-IA (*n* = 35) DAS 28 = 3.2–5.1	S-IA (*n* = 26) DAS-28 >5.1	ANOVA/chi-squared test
Mean age ± SD	46.41 ± 12.86	47 ± 8.75	52.4 ± 15.81	53.04 ± 17.16	0.466
Sex (% female)	0.41	0.4	0.51	0.73	0.146
Duration of the symptoms	2.37	1.5	2.11	3.98 ± 4.73	0.152
hsCRP (mean ± SE)	1.69 ± 0.383	2.86 ± 1.88	4.5 ± 1.11	15.48 ± 4.43	0.004
Median and IQR	1.3 (0.6, 2.6)	0 (0, 5)	2.3 (0.9, 4.9)	6.8 (3.55, 13.83)	
ESR (mean ± SE)	7.53 ± 1.22	11.6 ± 5.64	14.2 ± 1.58	31.44 ± 4.61	0.000
Median and IQR	5 (4, 11)	6 (6, 8)	11 (7, 22)	25 (19, 35)	
% RF positive	0.24	0	0.34	0.19	0.293
% ACPA positive	0.12	0.2	0.03	0.19	0.199
Number of joints with SH grade 1 (median and IQR)	0 (0, 0)	0 (0, 1)	0 (0, 2)	0 (0, 2)	0.929
Number of joints with SH grade 2 (median and IQR)	0 (0, 0)	0 (0, 0)	0 (0, 0)	0 (0, 0)	0.632
Number of joints with SH grade 3 (median and IQR)	0 (0, 0)	0 (0, 4)	0 (0, 3)	2.5 (0, 5.75)	0.023
Number of joints with PD (median and IQR)	0 (0, 0)	0 (0, 0)	0 (0, 0)	1 (0, 3)	0.000
Number of joints with osteophytes (median and IQR)	2 (0, 6)	3 (2, 5)	3 (1.5, 7)	4 (1, 6.75)	0.695
Number of joints with erosions (median and IQR)	0 (0, 0)	0 (0, 1)	0 (0, 0.5)	0 (0, 2)	0.096
% patients with PD	0.06	0	0.2	0.54	0.001
TJC (mean ± SD)	0.29 ± 0.59	1.8 ± 0.837	7.37 ± 6.10	18.19 ± 8.43	0.000
SJC (mean ± SD)	0.24 ± 0.75	0	2.28 ± 4.76	5.38 ± 6.82	0.006
GVAS (mean ± SD)	24.41 ± 22.35	55 ± 29.15	64 ± 19.73	73.46 ± 17.42	0.000
Pain VAS (mean ± SD)	36.43 ± 32.75	55 ± 25.17	62.22 ± 21.57	71.87 ± 23.15	0.023

**Table 4:** Comparison between RA and S-IA patients in clinical remission, with low, moderate or high disease activity based on the DAS-28 cutoffs

Disease activity	RA vs. S-IA (patients with DAS-28 <2.6) (*p* value)	RA vs. S-IA (patients with DAS-28 = 2.6–3.2) (*p* value)	RA vs. S-IA (patients with DAS-28 = 3.2–5.1)	RA vs. S-IA (patients with DAS-28 >5.1) (*p* value)
Mean age	0.67 (0.912)	−0.67 V (0.92)	5.77 (0.102)	4.32 (0.317)
Sex (% female)	0.254 (0.329)	0.156 (0.000)	0.156 (0.000)	–0.03 (0.999)
Duration of the symptoms	5.549 (0.004)	5.21 (0.168)	9.33 (0.00)	4.32 (0.02)
CRP	–0.13 (0.847)	0.773 (0.814)	0.98 (0.53)	–7.85 (0.078)
ESR	–0.029 (0.98)	–1.822 (0.738)	5.24 (0.13)	–1.107 (0.845)
RF positive	0.265 (0.28)	0.67 (0.064)	0.234 (0.054)	0.353 (0.013)
ACPA positive	0.382 (0.065)	0.356 (0.469)	0.395 (0.000)	0.353 (0.013)
Number of joints with SH grade 1	–0.09 (0.90)	1.578 (0.618)	2.34 (0.02)	1.26 (0.267)
Number of joints with SH grade 2	0.82 (0.268)	1.11 (0.478)	0.773 (0.125)	0.52 (0.238)
Number of joints with SH grade 3	0.941 (0.313)	0.09 (0.944)	1.08 (0.104)	–0.456 (0.628)
Number of joints with PD	0.608 (0.005)	0.33 (0.169)	1.92 (0.01)	0.682 (0.516)
Number of joints with osteophytes	0.686 (0.733)	1.47 (0.445)	0.226 (0.826)	–0.446 (0.734)
Number of joints with erosions	4.63 (0.003)	3.49 (0.047)	5.96 (0.000)	5.43 (0.000)
Number of patients with PD	0.44 (0.02)	0.33 (0.437)	0.43 (0.00)	0.128 (0.46)
TJC	0.706 (0.046)	0.756 (0.401)	–0.86 (0.475)	1.29 (0.544)
SJC	1.515 (0.290)	1.22 (0.189)	1.01 (0.252)	0.555 (0.72)
GVAS	–6.00 (0.502)	–20.56 (0.132)	–19.19 (0.000)	1.08 (0.815)
Pain VAS	–25 (0.09)	–13.00 (0.476)	–13.22 (0.08)	8.125 (0.2707)

Most importantly, the DAS-28 scores did not correlate significantly with the PD scores in the RA patients (*p* = 0.25, *p* = 0.87, *p* = 0.13, *p* = 0.22, respectively, for the remission, low, moderate and active diseases, respectively). However, in the S-IA group, DAS-28 scores above 5.1 correlated significantly with the PD score (*p* = 0.048).

The binominal model analysis evaluated the impact of several variables on the risk of having active joint inflammation assessed by the presence of PD. The most negative values suggested a lower risk for positive PD signal in correlation with a certain parameter. Table 5 summarises the marginal effects of different variables on the number of joints with PD at the US examination in S-IA, RA patients, and patients with other arthropathies (labelled as non S-IA and non-RA). For instance, the average PD scores of S-IA patients increased by 0.004856 units in our prediction model as a consequence of having a hsCRP level above rather than below 5 mg/l. Similarly, the average PD score of a patient diagnosed with RA and treated with tocilizumab decreased by 1.81 units as compared to an RA patient who is not treated with tocilizumab (in the context of similar clinical picture and laboratory results). The most striking finding was that RA patients treated with tocilizumab tended had a lower PD score on US examination than patients treated with other biologics or DMARDs, despite similar clinical and laboratory findings (Table 5). The presence of RF increased slightly the risk of positive PD score only in patients with S-IA, and ACPA was associated with higher risk of active disease in patients with established RA, but had less influence on the S-IA group. Out of all three clinical outcomes, TJC, SJC and GVAS, only the SJC correlated with an increased PD score and only in patients with RA. The effect of different variables on the risk for a higher PD score was close to a zero value in the case of treatment with MTX and etanercept. For example, our prediction model suggested that in this group, the RA patients treated with MTX or etanercept monotherapy had a similar risk of having active joint inflammation on US. In this particular case, all the outcome measures taken into consideration in the prediction model are more relevant than being treated with MTX rather than etanercept.

**Table 5:** Marginal effects of different variables on the number of joints with PD signal in patients with other arthropathies vs. S-IA vs. RA

RA status (*n* = 108)	0	0	1
Suspicion of IA (S-IA) status (*n* = 92)	0	1	0
RF	2.76 (2.32, 3.21)	0.11 (–0.25, 0.46)	–0.09 (–0.23, 0.06)
CCP	–0.75 (–1.07, –0.42)	0.01061559 (–0.35, 0.37)	0.65 (0.50, 0.80)
MTX	–	–	0.01 (–0.12, 0.13)
Etanercept	–	–	0.01 (–0.12, 0.13)
Rituximab	–	–	0.62 (0.45, 0.79)
Tocilizumab	–	–	–1.81 (–1.90, –1.72)
Adalimumab	–	–	0.79 (0.58, 1.01)
hsCRP (>5 mg/l)	0.0278 (0.0277, 0.0279)	0.004856 (0.004850, 0.004862)	0.0546 (0.0544, 0.0547)
ESR	0.01551 (0.01548, 0.01555)	0.01880 (0.01871, 0.01889)	0.007728 (0.007725, 0.007731)
TJC	–0.00915 (–0.00917, –0.00914)	0.02020 (0.02010, 0.02030)	–0.0018359 (–0.0018360,–0.0018357)
SJC	0.100 (0.099, 0.102)	0.086 (0.0841, 0.0879)	0.214 (0.212, 0.216)
GVAS	0.004594 (0.004590, 0.004597)	–0.01021 (–0.01024,–0.01019)	–0.005323 (–0.005324,–0.005321)

**Fig. 1:**
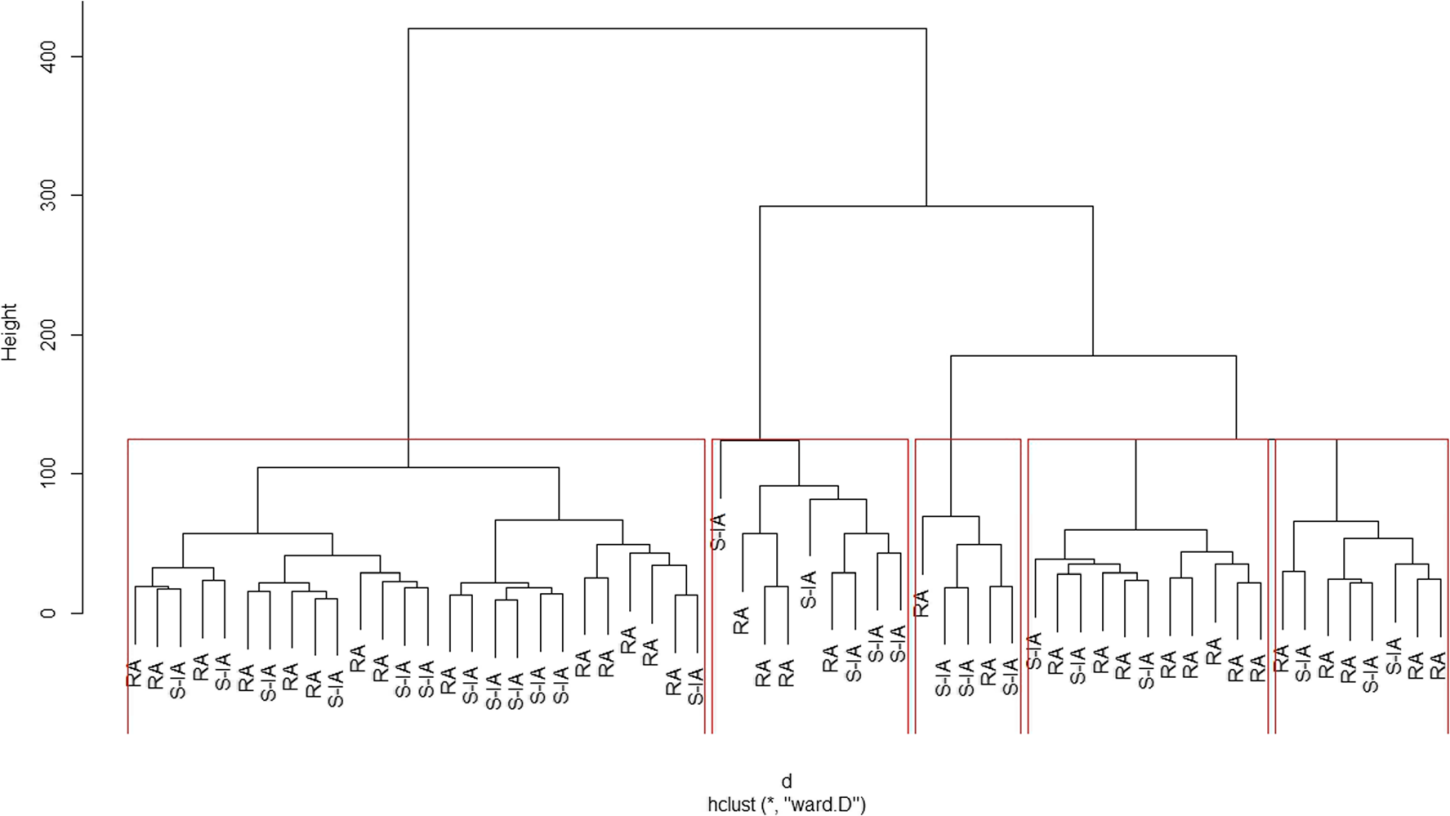
Dendogram showing the clustering of the patients with high disease activity score (DAS-28 >5.1) 336 × 204 mm (96 × 96 DPI)

We also attempted a cluster analysis of patients with similar DAS-28 scores using the following variables: age at scan; duration of symptoms; CRP; ESR; SH grades 1, 2 and 3; PD; presence of osteophytes; erosions; TJC; and SJC and GVAS. Although there was no obvious clustering of patients based on their diagnosis, the analysis revealed five big clusters with a large jump in the levels of two consecutive nodes (Fig. 1). One large cluster, including a significant proportion of S-IA patients with DAS-28 >5.1 exhibited the smallest amount of dissimilarity, suggesting that further analysis of larger patient groups might help identify which parameters are best to predict patients’ tendency to cluster in a certain disease group. Figure 1 represents the dendogram.

## Discussion

The results of our comparative analysis between the two groups of patients reflected their selection: patients with RA with no obvious clinical signs of active disease, but possible subclinical synovitis, and patients with S-IA, who did not fulfil the clinical and laboratory criteria for diagnosis of an inflammatory arthropathy and were referred for an US scan. As expected, the number of joints with active inflammation and clinical swelling was low. Patients with established RA had longer disease duration and more obvious chronic inflammatory changes (defined as SH and tendon inflammatory changes). Chronic joint changes characteristic for RA are known to pose clinical difficulty in assessing for the presence of active inflammation. As frequently encountered in clinical practice, patients with chronic conditions probably have better coping strategies, and this can account for the reported difference in the GVAS assessment between the two groups, despite similar pain scores.

A large proportion of our study patients with positive PD signal in at least one of their hand joints was diagnosed with a form of IA following the US scan (91.4 %, 43/47), which reiterates the role of US examination in the early diagnosis of IA. Also, our study showed that about one in two patients with inflammatory joint pains has active arthritis based on US examination, even if they have not been previously diagnosed with any joint disease. In addition, the DAS-28 score underestimated the presence of active inflammation in 50 % patients with RA classified as being in clinical remission; also these RA patients had more active disease, more erosions and joint tenderness that subjects with S-IA included in the same disease activity group.

The binominal regression analysis model found that certain parameters can have different impacts on the PD signal risk prediction. It is difficult to appreciate the magnitude of the effect of different parameters taken into consideration in this prediction model because of the limited number of patients included in our study. Tocilizumab is considered one of the most potent inhibitors of synovial inflammation and was expected to be associated with the lower risk for PD signal, even in the context of joint pains. The weak impact of SJC and TJC on the PD presence is a common finding of our prediction model and previous US studies. Our study also emphasised the limited role of the inflammatory markers in predicting joint inflammation, as we found that almost half of our RA patients with hsCRP below 5 mg/l had at least one joint with positive PD signal.

This is the first study to propose a statistical prediction model integrating clinical, laboratory and US parameters aiming to predict which characteristics are associated with an increased risk of having active joint inflammation at the US examination in two different groups of patients. The role of US scans in early detection of RA is well established [15], and the probability of developing clinically apparent arthritis in the context of positive PD signal was estimated at 94 % [16]. The main diagnosis difficulties arise when a patient does not fulfil criteria for a diagnosis of IA, and the availability of US scans is limited. Despite the effort to establish guidelines for early diagnosis of RA integrating US data [16], there are no algorithms to help with the exclusion of IA in the context of inflammatory hand pains. Ideally, all patients with inflammatory hand pain should have an US examination to exclude joint inflammation. The US examination was proven effective in altering treatment decision and had increased sensitivity compared to clinical examination [17].

Our real-life observational study confirmed that there were differences between patients with established RA and patients referred with suspicion of IA, in terms of parameters associated with the presence of PD in their joints and patients’ characteristics. As the patients were referred to have a scan because of the difficulty to appreciate if their joints had active inflammation or not, rather than being screened for US abnormalities in the context of arthritis, the two groups of patients are not representative for the general group of patients with IA and established RA.

It is interesting to note that the presence of SJ in patients with suspicion of IA did not correlate with the presence of PD at the US examination and that DAS-28 score is not able to discriminate between RA patients with more or less active joint inflammation on US.

It is recognised that patients with established RA have chronic inflammatory changes, and the presence of swelling is not always indicative of presence of active inflammation, whereas patients with no previous diagnosis of arthritis might get swellings if their joints become inflamed. However, previous studies found a disparity between the clinical assessment of joint swelling and tenderness and the presence of PD signal in patients with established RA [18]. In our study, the presence of PD did not correlate with the erosion scores in RA patients, probably because the two phenomena are temporarily distinct. Previous studies have been focused on patients with early RA, establishing the importance of US for early diagnosis [15], disease progression assessment [19] and prediction of risk to develop arthritis [3, 20]. The emphasis of our prediction model is rather on finding parameters that suggest the need for organising an US examination in certain categories of patients at risk, rather than establishing the patients’ risk of developing IA based on prospective serial US examinations, as the majority of studies in the literature.

Our study has some important limitations: the number of patients was too small for enabling definite conclusions in relation to the risk prediction model and no practical validation of this model was pursued as this was beyond the scope of this study. This prediction risk model, if validated, will ensure patient access to US scans based on the stratification of their risk of having sub-clinical joint inflammation to minimise the risk of under-diagnosing active RA in the context of limited NHS clinical resources. The US score used included only hand joints as the patients referred to our US clinics had hand joint inflammatory pains. We do not suggest the extrapolation of our study findings for other joint areas or for other inflammatory arthropathies. The distal interphalangeal joints were not examined on purpose, as they are not included in the RA OMERACT scores.

The performance of this prediction model improves with the expansion of the database and also by taking into consideration ‘missing US outcomes’. To address this, we plan to collect data in parallel from two RA patient groups (who had and had not a recent US scan) to optimise our model ability to predict sub-clinical inflammation in the absence of recorded US outcomes. A pilot study is also planned for the external validation of our risk model, in which we will compare the predicted model outcomes with the US data to assess our model performance.
